# Perspectives on How Human Simultaneous Multi-Modal Imaging Adds Directionality to Spread Models of Alzheimer’s Disease

**DOI:** 10.3389/fneur.2018.00026

**Published:** 2018-01-26

**Authors:** Julia Neitzel, Rachel Nuttall, Christian Sorg

**Affiliations:** ^1^Department of General and Experimental Psychology, Ludwig-Maximilians-Universität (LMU), München, Germany; ^2^TUM-Neuroimaging Center (TUM-NIC), Klinikum rechts der Isar, Technische Universität München (TUM), München, Germany; ^3^Department of Neuroradiology, Klinikum rechts der Isar, Technische Universität München (TUM), München, Germany; ^4^Department of Psychiatry and Psychotherapy, Klinikum rechts der Isar, Technische Universität München (TUM), München, Germany

**Keywords:** Alzheimer’s disease, spread of pathology, effective connectivity, metabolic connectivity mapping, simultaneous MR-PET imaging

## Abstract

Previous animal research suggests that the spread of pathological agents in Alzheimer’s disease (AD) follows the direction of signaling pathways. Specifically, tau pathology has been suggested to propagate in an infection-like mode along axons, from transentorhinal cortices to medial temporal lobe cortices and consequently to other cortical regions, while amyloid-beta (Aβ) pathology seems to spread in an activity-dependent manner among and from isocortical regions into limbic and then subcortical regions. These directed connectivity-based spread models, however, have not been tested directly in AD patients due to the lack of an *in vivo* method to identify directed connectivity in humans. Recently, a new method—metabolic connectivity mapping (MCM)—has been developed and validated in healthy participants that uses simultaneous FDG-PET and resting-state fMRI data acquisition to identify directed intrinsic effective connectivity (EC). To this end, postsynaptic energy consumption (FDG-PET) is used to identify regions with afferent input from other functionally connected brain regions (resting-state fMRI). Here, we discuss how this multi-modal imaging approach allows quantitative, whole-brain mapping of signaling direction in AD patients, thereby pointing out some of the advantages it offers compared to other EC methods (i.e., Granger causality, dynamic causal modeling, Bayesian networks). Most importantly, MCM provides the basis on which models of pathology spread, derived from animal studies, can be tested in AD patients. In particular, future work should investigate whether tau and Aβ in humans propagate along the trajectories of directed connectivity in order to advance our understanding of the neuropathological mechanisms causing disease progression.

## Introduction

Alzheimer’s disease (AD) is characterized by the extracellular accumulation of misfolded amyloid-β peptides (Aβ), i.e., Aβ plaques, and intracellular aggregates of hyperphosphorylated tau proteins, i.e., neurofibrillary tangles (NFTs) (1). With disease progression, Aβ plaques and NFTs increase in number, yet following distinct spatio-temporal trajectories as revealed by postmortem neuropathological investigations and molecular imaging (2–4). NFTs first emerge in the locus coeruleus and transentorhinal cortex—around the time when first symptoms arise—which subsequently spread to the hippocampus and other limbic regions before finally emerging in isocortical areas (2). Recent tau positron emission tomography (PET) imaging has largely confirmed this pattern of spread (4, 5). Conversely, several years before first symptom onset, Aβ plaques are initially found in the neocortex and subsequently spread to subcortical brain areas at advanced disease stages (6). This pattern has been essentially replicated by amyloid-PET imaging (3).

**Figure 1:**
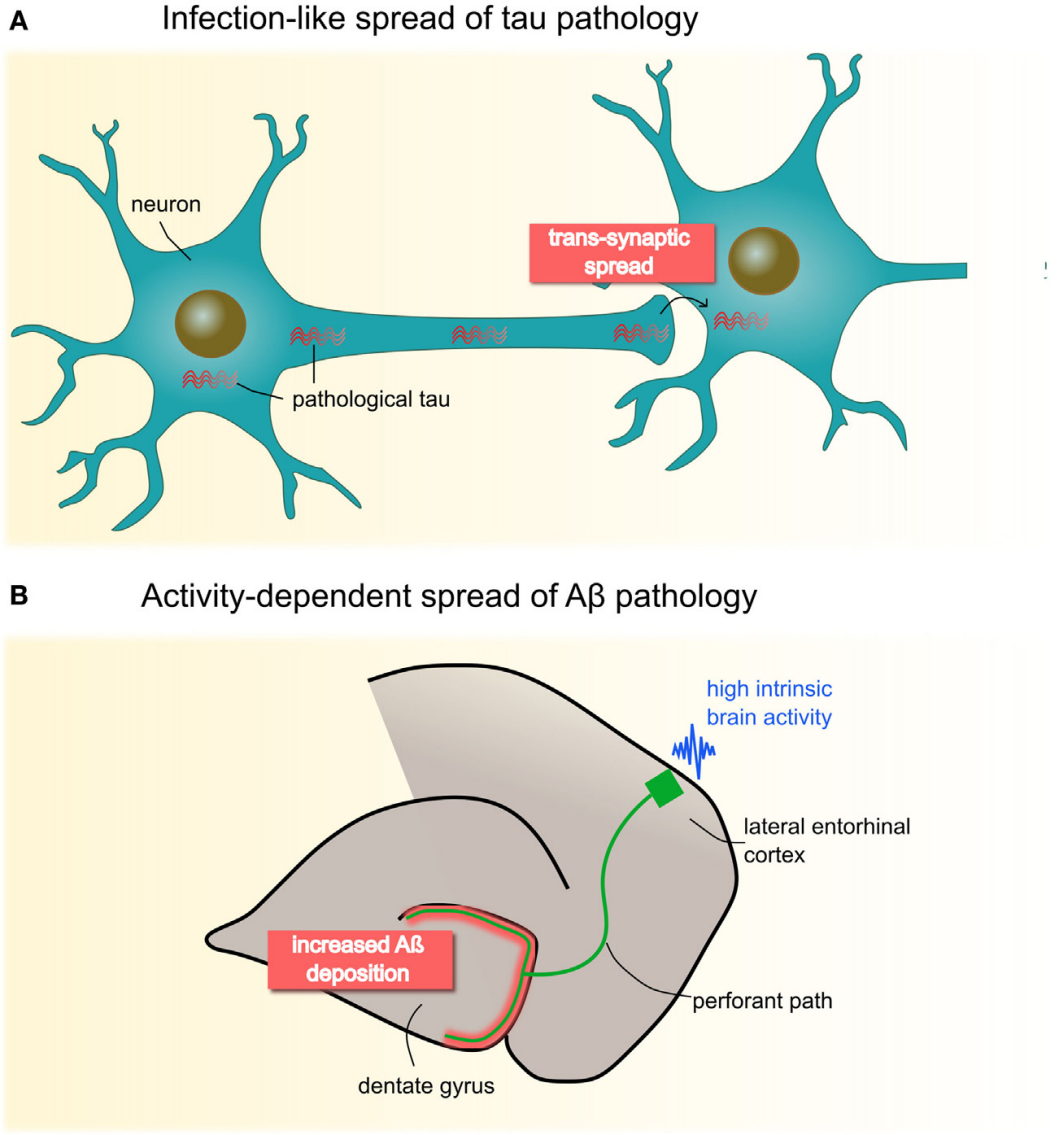
Animal studies have proposed two molecular mechanisms of neuropathological spread in Alzheimer’s disease **(A)** Pathological tau seems to propagate in an infectious- or prion-like mode: fibrillary protein seeds travel through the axon and across synapses to healthy cells, where they induce template-directed misfolding and aggregation of, until then, naïve proteins. Seminal work by Clavaguera et al. (8) shows that injections of brain extracts from a transgenic mouse line expressing mutant human tau induces misfolding of endogenous tau in recipient mice. Notably, over time, tau aggregates were found beyond the injection site in remote brain areas pointing to a self-propagating, trans-synaptic spread mechanism. **(B)** Deposition of Aβ has been shown to occur in an activity-dependent manner, such that chronic synaptic hyperactivity, e.g., in highly connected brain regions, is causally related to Aβ burden. This has been convincingly demonstrated by Yamamoto et al. (12) who applied chronic optogenetic activation of the hippocampal perforant pathway in a transgenic mice line expressing the amyloid β precursor protein. Their data revealed that optic stimulation of the lateral entorhinal cortex over 5 months heightens Aβ deposition specifically in presynaptic projection areas (i.e., dental gyrus), possibly though induced hyperactivity. Panel **(B)** is modified from Yamamoto et al. (12), open access article under the CC BY license (http://creativecommons.org/licenses/by/4.0/).

Animal models suggest that the spread of these pathologies depends critically on the directionality of neural connections (7). Figure 1 provides a schematic illustration of these spreading processes. Tau pathology appears to disseminate in an infection-like or prion-like fashion, whereby a self-propagating “infectious” tau protein emerges in intracellular compartments, spreads along the axon, and trans-synaptically induces pathological changes in nearby normal counterparts (8, 9). Aβ has been suggested to spread in an activity-dependent manner: Aβ aggregates trigger aberrant synaptic activity, resulting in hyperactivity (10, 11), which in turn induces increasing rates of Aβ pathology in remote but directly connected regions *via* axons, likely *via* induced hyperactivity (12).

Neuroimaging has further delineated these spreading pathways in humans (5, 13–16) showing that a regions vulnerability to pathological changes depends on connectivity strength, rather than proximity, to the initially affected areas. Myers et al. (13) found that areas with high functional connectivity (FC) during rest, especially the posterior default mode network (DMN), were associated with higher Aβ burden using a within-subject spatial correlation approach. However, a more direct link between pathology spread and directed connectivity—as suggested by animal models—have not been established in AD patients due to the lack of methods to identify directed connectivity pathways in humans.

Developments in simultaneous multi-modal imaging now offer new approaches to investigate directional signaling, or effective connectivity (EC), *in vivo*. Specifically, a new measure of intrinsic EC (iEC) has recently been established that exploits the simultaneous acquisition of energy metabolism and FC measures on a hybrid MR-PET scanner (17). In this paper, we discuss how this new approach adds a novel quantitative measure of spreading directionality in AD patients.

### Metabolic Connectivity Mapping (MCM) Provides a Measure of Signaling Directionality in AD Patients

Numerous studies have used undirected FC, defined as statistical dependencies between the activity signals of two brain regions, to investigate pathways of pathology propagation [e.g., Ref. (16)]. However, correlation analyses do not provide information on the influence that one region exerts over another. To understand the signaling hierarchy across distributed networks of regions, measures of EC, i.e., the directed, causal, activity-dependent relationship between regions, are usually more insightful (18).

**Figure 2:**
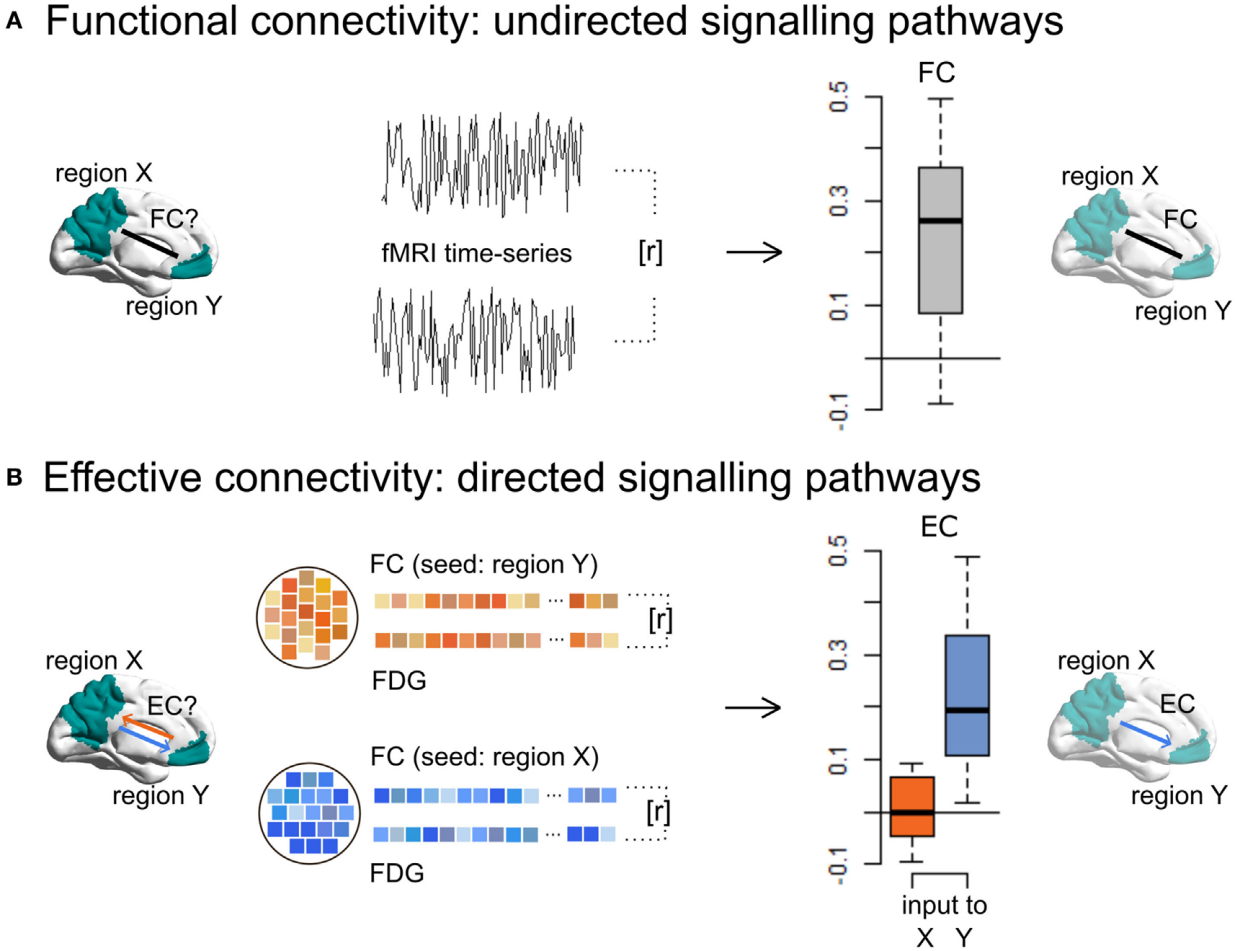
Metabolic connectivity mapping identifies intrinsic effective connectivity (EC), a proxy for directionality of signaling, in the human brain. **(A)** By using resting-state fMRI only, functional connectivity (FC), i.e., temporal correlations (*r*) between the spontaneous blood-oxygen-level-dependent fluctuations of a cluster X and Y, reflects non-directional communication among macroscopic brain regions. **(B)** Simultaneously acquired fMRI and FDG-PET data allow for the estimation of EC, i.e., the voxel-wise correlations (*r*) of FC and energy consumption. Since cellular recordings (see text) showed that the majority of signaling-related energy is consumed postsynaptically, positive correlations in a given region indicate signaling input along a FC pathway. This novel method can detect disease-related changes in directed connectivity in Alzheimer’s disease patients and further allows one to test directed connectivity-based spread models suggested by animal research. This figure is modified from Riedl et al. (17).

A novel approach to identify EC in humans integrates undirected FC with local energy consumption based on simultaneously acquired ^18^F-fludeoxyglucose (FDG) PET and resting-state functional magnetic resonance imaging (17). This method, called “metabolic connectivity mapping,” reveals ongoing or iEC (Figure 2). The underlying principle of this method is that most energy is spent on signaling processes, 75% of which is consumed postsynaptically (19). At the macroscopic level, it can be assumed that an increase in local metabolism reflects an increase in afferent EC from source regions. In more detail, the directionality of a single functional pathway linking two regions is investigated by taking the cluster FC time series for one region (the potential seed region), which is correlated with the time series of each voxel in another region (the potential target region), reaping one score of FC for each voxel in the target region. On a voxel-wise level, these scores of FC are then correlated with FDG activity, which, if correlated, infer that this region is the target of this functional pathway. If the same analysis with the seed and target regions switched also shows a significant correlation between FC and FDG values, this is a bidirectional pathway. This analysis is repeated for all region pairs, resulting in a voxel-wise, whole-brain mapping of EC.

Riedl and colleagues (17) have already applied this method to infer the healthy signaling hierarchy in states of externally directed attention (eyes open condition) versus internally directed attention (eyes closed condition). The authors observed bidirectional communication between early and higher visual areas of occipito-parietal lobes plus top-down signaling from a frontoparietal “dorsal attention” network, independent of condition. As soon as participants opened their eyes, parts of the salience network (including insular and cingulate cortices) exert additional top-down influences on the calcarine sulcus. These data support the idea that MCM reveals dynamically changing signaling pathways and, critically, captures the direction of communication among neural networks.

### Looking at Other Methods to Infer EC in Humans, Their Application to AD, and Methodological Issues

Other researchers have used statistical approaches to infer EC from undirected fMRI data, including Granger causality mapping (GCM) (20, 21), dynamic causal modeling (DCM) (22), and Bayesian network (BN) learning (23). These methods have been used to investigate changed network dynamics in AD patients, reporting disrupted EC in the DMN, though with certain caveats.

Granger causality mapping is based on the assumption that causes precede, and help to predict, consequences. Vector autoregressive models are used to analyze causal interactions between two brain regions, in which the blood-oxygen-level-dependent (BOLD) signal of one region Y at a particular time is modeled as a linear weighted sum of its own past BOLD fluctuations and that of another region X. Activity in area X is said to “Granger” cause activity in area Y if the past of X contains information that helps to predict the future of Y, over and above the information already in the past of Y itself [for review, see Ref (24)]. Applied to AD, GCM revealed altered EC among DMN regions. While the connection strength to the posterior cingulate cortex was markedly reduced in AD patients compared to healthy controls, the medial prefrontal cortex showed stronger coupling with bilateral inferior parietal regions (25). Contrasting results were found by another GCM study reporting relatively preserved posterior cingulate cortex connectivity in AD patients (26). Disease-related changes in GCM have also been found in other networks besides the DMN, e.g., in the executive control network (27). Notably, several assumptions underlie the application of linear autoregressive models to fMRI. While a detailed account can be found in Ref. (28), the strongest criticism that has been raised concerns spurious “causality” that is in fact the result of naturally occurring time-lags among different brain regions. For example, GCM applied to simulated fMRI time-series data was shown to perform relatively poorly, which “suggests that the directionality results may not be trustworthy” (29).

In contrast to GCM, DCM does not estimate EC directly from the observed activity among different brain regions, but instead infers causality from hidden (unobserved) neuronal states that cause those observations. These hidden states are described in terms of bilinear differential equations, which define how the present state of a particular region influences the dynamics of another under experimental manipulation. In order to infer causal interactions at the neural level, DCM integrates a hemodynamic forward model that describes the transformation from neural activity to the measured BOLD signal. Finally, a Bayesian model selection is used to identify the most likely among a set of competing DCMs by comparing the probability of observing the data under a particular model [for technical details, see Ref (30)]. Up to now, only one research group implemented DCM in AD patients (31). In this work, strength of EC was computed during a simple motor task. Compared to healthy control participants, AD patients had significantly reduced EC between the left and right primary somato-motor cortices. The relative lack of DCM studies in the AD literature might be attributed to some restrictions inherent to this approach. The most fundamental issue is that the assumptions held by the hemodynamic forward model, i.e., the mapping between neuronal activity (hidden states) and measured BOLD response, are most probably violated in AD patients due to the damaged vasculature. In brief, neuronal activity drives vasodilation and thereby increases blood flow, which inflates blood volume and reduces the concentration of deoxyhemoglobin. The latter enters the hemodynamic response equation [for more details, see Ref. (32)]. A growing body of evidence indicates that Aβ not only effects neurons but also cerebral blood vessels (33, 34). Decreased arterial blood flow has been found in healthy old carriers of the APOE ε4 genotype, individuals with mild cognitive impairment and AD patients [reviewed by Zhang et al. (35)]. Consequently, the interpretation of DCM results obtained within the AD spectrum is less straightforward; reduced EC could point to altered neuronal interactions and/or AD-related changes in the neurovascular coupling. Furthermore, parameter estimates are wholly dependent on which set of brain regions are included in the DCM, since it is neither mathematically nor computationally feasible to efficiently search over the full range of all possible regions. Therefore, the resulting patterns of EC are only a parsimonious model of the “real” causal architecture. The problem of missing or novel nodes not considered in the predefined model could be quite serious in AD, where atrophy might profoundly alter inter-regional connectivity (36).

Unlike the aforementioned EC methods, BN approaches aim to train a suitable EC model from the data alone, without the need for prior knowledge and considering the entire brain (23). A BN modal is a directed acyclic (no loops that start or end at the same node) graph that consists of nodes representing neuronal regions and edges that symbolize inter-region connectivity. Conditional probability densities are used to determine the functional network structure. BN-inferred EC patterns of AD patients show a global disruption of connectivity from the hippocampus to other main hubs of the DMN, e.g., to the posterior cingulate and medial prefrontal cortex, while coupling between left and right hippocampi were abnormally increased in patients compared to controls (37). Despite the advantages that BN methods encompass compared to other network modeling techniques, the test results obtained from Smith et al.’s simulation study is not all positive (29). Although BN methods were found to excellently detect network connections, estimated directionality was close to chance performance. One restriction in this respect is that BN cannot model reciprocal connections.

### MCM Can Capture the Spread of Pathology in Whole-Brain and Quantitative Terms

We propose that MCM is a promising new tool that, based on the benefits of multi-modal MR-PET imaging, allows one not only to map iEC changes in AD patients but also to link such changes with pathology spread. Especially in the context of AD, this approach offers several advantages compared to other EC methods. First, MCM is a data-driven or model-free approach which requires comparably little pre-assumptions. In fact, unidirectional as well as reciprocal connections can be captured between any regions spanning the whole brain. This is a favorable property considering that little is known about how AD targets the EC structure of the human brain. Second, MCM is less error-prone to naturally occurring as well as AD-related, inter-regional variations in the neurovascular coupling that cause inhomogeneity in the measured BOLD signal. The reason for that is, EC is not directly estimated from the BOLD response, but from correlating the BOLD time series between distinct regions making it invariant to the signal amplitude. In terms of between-subject variations, Riedl and colleagues (17) showed that MCM can reveal robust and condition-specific changes of EC in a group of healthy participants. Thus, the authors concluded “that the assessment of changes in EC may be more robust to vascular heterogeneity.” Third, capturing signal directionality from two imaging modalities with similar voxel size also has distinct advantages regarding sensitivity. Since the data are collected simultaneously and independently from the same patient, preprocessing steps commonly applied before statistical analyses, which spatially distort the data, e.g., spatial normalization and smoothing, can be omitted. Instead, the new approach allows EC mapping in individual subject space and may be even sensitive for single-subject analyses. A final, practical advantage of MCM is that EC can be assessed during the resting state, free of any cognitive demand. Mapping iEC opens up novel opportunities for linking the brain’s endogenous signaling hierarchy in AD patients with molecular theories about pathology propagation for which experimental evidence has as of yet been restricted to animal models.

Despite being a highly promising method, it is important to highlight the limitations of MCM. First, there is a large difference between FDG-PET imaging and fMRI in terms of temporal resolution: the former can only acquire one saturated image after a period of 30-min scanning, which can cause problems when analyzed in conjunction with a relatively temporally precise and dynamic measure such as fMRI-based FC (38). It is important to adopt a study design that measures stable FC across extended periods when using MCM, so as to ensure similar time scales across both imaging modalities (17).

Second, vascular heterogeneity in terms of vascular density and cerebral blood flow has been shown to influence BOLD-FC (39–41), which can lead to spatial inhomogeneities in the measured BOLD signals and hence may induce false-positives/negatives in the spatial FDG-FC voxel-wise correlations. However, as mentioned previously, since MCM utilizes FC rather than the BOLD signal directly, concern over this potential limitation is somewhat reduced (17).

Finally, one must keep in mind that MCM can only obtain a proxy of EC, since it uses energy consumption as an indication of signaling direction. Recent studies have shown strong support for the underlying assumption that energy consumption is mostly conducted directly at neurons (42), but the findings for a possible role of astrocytes in glucose uptake suggest that the underlying mechanisms of neuroenergetics may not be so clear cut (43). The BOLD signal is also a proxy measure of neuronal activity, but the neuronal basis of the BOLD signal has been widely supported (44–46). The established drawbacks of PET in terms of resolution and sensitivity and its utility in the study of AD pathology should also be taken into consideration when applying MCM to investigate EC and spread models of AD pathology, which have been extensively discussed in other articles (47–52). Additionally, other multi-modal imaging techniques such as fMRI with MR spectroscopy or flumazenil-PET may also offer interesting insight into AD pathology and FC [for reviews, see Ref (53–55)] but, unlike FDG-PET/fMRI, they do not yet offer the key aspect of directionality of functional pathways, along which animal models have shown amyloid-β and tau pathology to spread (7).

### Application of MCM in AD Patients and Other Neurodegenerative Conditions

Specific approaches to testing spread models of pathology are outlined below. The general logic of these approaches is to compare maps of pathology characteristics, derived from imaging AD patients, and maps of iEC characteristics and changes in these maps in pre-stage AD patients, such as mild cognitive impairment or subjective cognitive impairment. On the one hand, PET-based pathology imaging has demonstrated significant amounts of pathology in these pre-stage AD patients, on the other hand, FC, which forms the basis of EC is largely preserved, facilitating reliable EC. The ultimate question, then, would be to what extent the pathology patterns can be explained by EC pathways. As a simple example, we suggest that, for a pair of regions sharing intact unidrectional EC and a significant gradient of pathology, some variance in this pathology gradient across patients can be explained by variance in the strength of EC beyond underlying functional or structural connectivity. A further example might be a longitudinal approach, in which the increase of a region’s pathology is explained by iEC into this region at the time of first measurement.

Furthermore, the application of MCM to the investigation of other neurodegenerative conditions seems promising. Despite their clinical heterogeneity, many neurodegenerative diseases share a common neurological signature—the misfolding and accumulation of specific proteins. Besides AD, this is the case in Parkinson’s disease characterized by α-synuclein; sporadic amyotrophic lateral sclerosis and rare fronto-temporal dementia showing aggregates of TAR DNA-binding protein 43 or in Huntington’s disease with huntingtin aggregates. Cell culture and/or animal studies more and more firmly demonstrate that these misfolded proteins share the ability of self-perpetuating neuron-to-neuron spreading, implying that neuronal connections probably play a critical role in disease propagation [see Ref (7, 56, 57), for recent reviews]. First evidence for a direction-dependent spreading mechanism have been particularly shown for α-synuclein. Pathological changes in Parkinson’s disease appear in a prototypical sequence starting in the lower brainstem and olfactory bulb, from where they proceed to the midbrain and the substantia nigra, before being found in the basal forebrain and ultimately in the neocortex (58). Moreover, α-synuclein’s ability to propagate transneuronally along defined neuronal pathways has been confirmed in transgenic mice. After intracerebral injection of brain-derived, pathological α-synuclein, the asymptomatic recipient animals developed Parkinson’s disease-like lesions which were also observed in interconnected regions far beyond the injection sites (59). Estimating direction of neuronal communication in humans by MCM may hence allow testing such an infection-like spreading model in Parkinson’s disease patients.

## Conclusion

Better knowledge of the mechanisms that cause propagation of Aβ and tau pathology from an initially isolated target site to remote regions of wider brain networks will pave the way for more precise diagnostics and novel treatment strategies. Given the clear predictions of animal models that AD pathology spreads in the direction of neuronal pathways, future research should aim to explicitly test this idea in AD patients. MCM has been demonstrated to be a capable tool for detecting iEC, a proxy for directed connectivity, in healthy participants. Applied to AD patients, this multi-modal imaging approach allows future studies to test whether the spread of tau and Aβ pathology in humans follows the hypothesized trajectories of iEC.

## Author Contributions

All authors contributed to the conceptualization and the writing of the article.

## Conflict of Interest Statement

The authors declare that the research was conducted in the absence of any commercial or financial relationships that could be construed as a potential conflict of interest. The reviewer VT and handling editor declared their shared affiliation.

## References

[1] Nelson PT Alafuzoff I Bigio EH Bouras C Braak H Cairns NJ Correlation of Alzheimer disease neuropathologic changes with cognitive status: a review of the literature. J Neuropathol Exp Neurol (2012) 71(5):362–81. 10.1097/NEN.0b013e31825018f7 22487856 PMC3560290

[2] Braak H Braak E. Neuropathological stageing of Alzheimer-related changes. Acta Neuropathol (1991) 82(4):239–59. 10.1007/BF00308809 1759558

[3] Klunk WE Engler H Nordberg A Wang Y Blomqvist G Holt DP Imaging brain amyloid in Alzheimer’s disease with Pittsburgh Compound-B. Ann Neurol (2004) 55(3):306–19. 10.1002/ana.20009 14991808

[4] Schöll M Lockhart SN Schonhaut DR O’Neil JP Janabi M Ossenkoppele R PET imaging of tau deposition in the aging human brain. Neuron (2016) 89(5):971–82. 10.1016/j.neuron.2016.01.028 26938442 PMC4779187

[5] Sepulcre J Schultz AP Sabuncu M Gomez-Isla T Chhatwal J Becker A In vivo tau, amyloid, and gray matter profiles in the aging brain. J Neurosci (2016) 36(28):7364–74. 10.1523/JNEUROSCI.0639-16.2016 27413148 PMC4945661

[6] Thal DR Rüb U Orantes M Braak H Phases of Aβ-deposition in the human brain and its relevance for the development of AD. Neurology (2002) 58(12):1791–800. 10.1212/WNL.58.12.1791 12084879

[7] Brettschneider J Del Tredici K Lee VM Trojanowski JQ. Spreading of pathology in neurodegenerative diseases: a focus on human studies. Nat Rev Neurosci (2015) 16(2):109–20. 10.1038/nrn3887 25588378 PMC4312418

[8] Clavaguera F Bolmont T Crowther RA Abramowski D Frank S Probst A Transmission and spreading of tauopathy in transgenic mouse brain. Nat Cell Biol (2009) 11(7):909–13. 10.1038/ncb1901 19503072 PMC2726961

[9] Frost B Diamond MI. Prion-like mechanisms in neurodegenerative diseases. Nat Rev Neurosci (2010) 11(3):155–9. 10.1038/nrn2786 20029438 PMC3648341

[10] Busche MA Eichhoff G Adelsberger H Abramowski D Wiederhold K Haass C Clusters of hyperactive neurons near amyloid plaques in a mouse model of Alzheimer’s disease. Science (2008) 321(5896):1686–9. 10.1126/science.1162844 18802001

[11] Palop JJ Mucke L Amyloid-[beta]-induced neuronal dysfunction in Alzheimer’s disease: from synapses toward neural networks. Nat Neurosci (2010) 13(7):812–8. 10.1038/nn.2583 20581818 PMC3072750

[12] Yamamoto K Tanei Z Hashimoto T Wakabayashi T Okuno H Naka Y Chronic optogenetic activation augments Aβ pathology in a mouse model of Alzheimer disease. Cell Rep (2015) 11(6):859–65. 10.1016/j.celrep.2015.04.017 25937280

[13] Myers N Pasquini L Göttler J Grimmer T Koch K Ortner M Within-patient correspondence of amyloid-β and intrinsic network connectivity in Alzheimer’s disease. Brain (2014) 137(7):2052–64. 10.1093/brain/awu103 24771519 PMC4065018

[14] Pasquini L Benson G Grothe MJ Utz L Myers NE Yakushev I Individual correspondence of amyloid-β and intrinsic connectivity in the posterior default mode network across stages of Alzheimer’s disease. J Alzheimers Dis (2017) 58(3):763–73. 10.3233/JAD-170096 28482640

[15] Raj A Kuceyeski A Weiner M. A network diffusion model of disease progression in dementia. Neuron (2012) 73(6):1204–15. 10.1016/j.neuron.2011.12.040 22445347 PMC3623298

[16] Zhou J Gennatas ED Kramer JH Miller BL Seeley WW. Predicting regional neurodegeneration from the healthy brain functional connectome. Neuron (2012) 73(6):1216–27. 10.1016/j.neuron.2012.03.004 22445348 PMC3361461

[17] Riedl V Utz L Castrillón G Grimmer T Rauschecker JP Ploner M Metabolic connectivity mapping reveals effective connectivity in the resting human brain. Proc Natl Acad Sci U S A (2016) 113(2):428–33. 10.1073/pnas.1513752113 26712010 PMC4720331

[18] Friston KJ. Functional and effective connectivity: a review. Brain Connect (2011) 1(1):13–36. 10.1089/brain.2011.0008 22432952

[19] Harris JJ Jolivet R Attwell D. Synaptic energy use and supply. Neuron (2012) 75(5):762–77. 10.1016/j.neuron.2012.08.019 22958818

[20] Goebel R Roebroeck A Kim D Formisano E. Investigating directed cortical interactions in time-resolved fMRI data using vector autoregressive modeling and Granger causality mapping. J Magn Reson Imaging (2003) 21(10):1251–61. 10.1016/j.mri.2003.08.026 14725933

[21] Harrison L Penny WD Friston K. Multivariate autoregressive modeling of fMRI time series. Neuroimage (2003) 19(4):1477–91. 10.1016/S1053-8119(03)00160-5 12948704

[22] Friston KJ Harrison L Penny W. Dynamic causal modelling. Neuroimage (2003) 19(4):1273–302. 10.1016/S1053-8119(03)00202-7 12948688

[23] Zheng X Rajapakse JC. Learning functional structure from fMR images. Neuroimage (2006) 31(4):1601–13. 10.1016/j.neuroimage.2006.01.031 16540348

[24] Seth AK Barrett AB Barnett L Granger causality analysis in neuroscience and neuroimaging. J Neurosci (2015) 35(8):3293–7. 10.1523/JNEUROSCI.4399-14.2015 25716830 PMC4339347

[25] Zhong Y Huang L Cai S Zhang Y von Deneen KM Ren A Altered effective connectivity patterns of the default mode network in Alzheimer’s disease: an fMRI study. Neurosci Lett (2014) 578:171–5. 10.1016/j.neulet.2014.06.043 24996191 PMC6293460

[26] Miao X Wu X Li R Chen K Yao L Altered connectivity pattern of hubs in default mode network with Alzheimer’s disease: an Granger causality modeling approach. PLoS One (2011) 6(10):e25546 10.1371/journal.pone.0025546 22022410 PMC3191142

[27] Liu Z Zhang Y Bai L Yan H Dai R Zhong C Investigation of the effective connectivity of resting state networks in Alzheimer’s disease: a functional MRI study combining independent components analysis and multivariate Granger causality analysis. NMR Biomed (2012) 25(12):1311–20. 10.1002/nbm.2803 22505275

[28] Valdes-Sosa PA Roebroeck A Daunizeau J Friston K. Effective connectivity: influence, causality and biophysical modeling. Neuroimage (2011) 58(2):339–61. 10.1016/j.neuroimage.2011.03.058 21477655 PMC3167373

[29] Smith SM Miller KL Salimi-Khorshidi G Webster M Beckmann CF Nichols TE Network modelling methods for FMRI. Neuroimage (2011) 54(2):875–91. 10.1016/j.neuroimage.2010.08.063 20817103

[30] Stephan KE Penny WD Moran RJ den Ouden HE Daunizeau J Friston KJ. Ten simple rules for dynamic causal modeling. Neuroimage (2010) 49(4):3099–109. 10.1016/j.neuroimage.2009.11.015 19914382 PMC2825373

[31] Agosta F Rocca MA Pagani E Absinta M Magnani G Marcone A Sensorimotor network rewiring in mild cognitive impairment and Alzheimer’s disease. Hum Brain Mapp (2010) 31(4):515–25. 10.1002/hbm.20883 19777557 PMC6871105

[32] Stephan KE Weiskopf N Drysdale PM Robinson PA Friston KJ. Comparing hemodynamic models with DCM. Neuroimage (2007) 38(3):387–401. 10.1016/j.neuroimage.2007.07.040 17884583 PMC2636182

[33] Park L Anrather J Zhou P Frys K Pitstick R Younkin S NADPH oxidase-derived reactive oxygen species mediate the cerebrovascular dysfunction induced by the amyloid β peptide. J Neurosci (2005) 25(7):1769–77. 10.1523/JNEUROSCI.5207-04.2005 15716413 PMC6725936

[34] Zlokovic BV Neurovascular pathways to neurodegeneration in Alzheimer’s disease and other disorders. Nat Rev Neurosci (2011) 12(12):723–38. 10.1038/nrn3114 22048062 PMC4036520

[35] Zhang N Gordon ML Goldberg TE Cerebral blood flow measured by arterial spin labeling MRI at resting state in normal aging and Alzheimer’s disease. Neurosci Biobehav Rev (2017) 72:168–75. 10.1016/j.neubiorev.2016.11.023 27908711

[36] Seghier ML Zeidman P Neufeld NH Leff AP Price CJ. Identifying abnormal connectivity in patients using dynamic causal modeling of FMRI responses. Front Syst Neurosci (2010) 4(142):1–14. 10.3389/fnsys.2010.00142 20838471 PMC2936900

[37] Wu X Li R Fleisher AS Reiman EM Guan X Zhang Y Altered default mode network connectivity in Alzheimer’s disease—a resting functional MRI and Bayesian network study. Hum Brain Mapp (2011) 32(11):1868–81. 10.1002/hbm.21153 21259382 PMC3208821

[38] Allen EA Damaraju E Plis SM Erhardt EB Eichele T Calhoun VD. Tracking whole-brain connectivity dynamics in the resting state. Cereb Cortex (2014) 24(3):663–76. 10.1093/cercor/bhs352 23146964 PMC3920766

[39] Liang X Zou Q He Y Yang Y. Coupling of functional connectivity and regional cerebral blood flow reveals a physiological basis for network hubs of the human brain. Proc Natl Acad Sci U S A (2013) 110(5):1929–34. 10.1073/pnas.1214900110 23319644 PMC3562840

[40] Vigneau-Roy N Bernier M Descoteaux M Whittingstall K Regional variations in vascular density correlate with resting-state and task-evoked blood oxygen level-dependent signal amplitude. Hum Brain Mapp (2014) 35(5):1906–20. 10.1002/hbm.22301 23843266 PMC6869285

[41] Liu J Hao Y Du M Wang X Zhang J Manor B Quantitative cerebral blood flow mapping and functional connectivity of postherpetic neuralgia pain: a perfusion fMRI study. Pain (2013) 154(1):110–8. 10.1016/j.pain.2012.09.016 23140909

[42] Lundgaard I Li B Xie L Kang H Sanggaard S Haswell JD Direct neuronal glucose uptake heralds activity-dependent increases in cerebral metabolism. Nat Commun (2015) 6:6807. 10.1038/ncomms7807 25904018 PMC4410436

[43] Zimmer ER Parent MJ Souza DG Leuzy A Lecrux C Kim HI [18F] FDG PET signal is driven by astroglial glutamate transport. Nat Neurosci (2017) 20(3):393–5. 10.1038/nn.4492 28135241 PMC5378483

[44] Logothetis NK Pauls J Augath M Trinath T Oeltermann A. Neurophysiological investigation of the basis of the fMRI signal. Nature (2001) 412(6843):150–7. 10.1038/35084005 11449264

[45] Logothetis NK The neural basis of the blood-oxygen-level-dependent functional magnetic resonance imaging signal. Philos Trans R Soc of Lond B Biol Sci (2002) 357(1424):1003–37. 10.1098/rstb.2002.1114 12217171 PMC1693017

[46] Viswanathan A Freeman RD. Neurometabolic coupling in cerebral cortex reflects synaptic more than spiking activity. Nat Neurosci (2007) 10(10):1308–12. 10.1038/nn1977 17828254

[47] Müller-Gärtner HW Links JM Prince JL Bryan RN McVeigh E Leal JP Measurement of radiotracer concentration in brain gray matter using positron emission tomography: MRI-based correction for partial volume effects. J Cereb Blood Flow Metab (1992) 12(4):571–83. 10.1038/jcbfm.1992.81 1618936

[48] Su Y Blazey TM Snyder AZ Raichle ME Marcus DS Ances BM Partial volume correction in quantitative amyloid imaging. Neuroimage (2015) 107:55–64. 10.1016/j.neuroimage.2014.11.058 25485714 PMC4300252

[49] Buchbender C Hartung-Knemeyer V Forsting M Antoch G Heusner TA Positron emission tomography (PET) attenuation correction artefacts in PET/CT and PET/MRI. Br J Radiol (2013) 86(1025):20120570 10.1259/bjr.20120570 23580397 PMC3635797

[50] Ossenkoppele R Schonhaut DR Schöll M Lockhart SN Ayakta N Baker SL Tau PET patterns mirror clinical and neuroanatomical variability in Alzheimer’s disease. Brain (2016) 139(5):1551–67. 10.1093/brain/aww027 26962052 PMC5006248

[51] Mishra S Gordon BA Su Y Christensen J Friedrichsen K Jackson K AV-1451 PET imaging of tau pathology in preclinical Alzheimer disease: defining a summary measure. Neuroimage (2017) 161:171–8. 10.1016/j.neuroimage.2017.07.050 28756238 PMC5696044

[52] Doraiswamy PM Sperling RA Coleman RE Johnson KA Reiman EM Davis MD Amyloid-β assessed by florbetapir F 18 PET and 18-month cognitive decline a multicenter study. Neurology (2012) 79(16):1636–44. 10.1212/WNL.0b013e3182661f74 22786606 PMC3468774

[53] Nava-Mesa MO Jiménez-Díaz L Yajeya J Navarro-Lopez JD GABAergic neurotransmission and new strategies of neuromodulation to compensate synaptic dysfunction in early stages of Alzheimer’s disease. Front Cell Neurosci (2014) 8(167):1–19. 10.3389/fncel.2014.00167 24987334 PMC4070063

[54] Li Y Sun H Chen Z Xu H Bu G Zheng H Implications of GABAergic neurotransmission in Alzheimer’s disease. Front Aging Neurosci (2016) 8(31):1–12. 10.3389/fnagi.2016.00031 26941642 PMC4763334

[55] Teipel S Drzezga A Grothe MJ Barthel H Chételat G Schuff N Multimodal imaging in Alzheimer’s disease: validity and usefulness for early detection. Lancet Neurol (2015) 14(10):1037–53. 10.1016/S1474-4422(15)00093-9 26318837

[56] Eraña H Venegas V Moreno J Castilla J. Prion-like disorders and transmissible spongiform encephalopathies: an overview of the mechanistic features that are shared by the various disease-related misfolded proteins. Biochem Biophys Res Commun (2017) 483(4):1125–36. 10.1016/j.bbrc.2016.08.166 27590581

[57] Stopschinski BE Diamond MI. The prion model for progression and diversity of neurodegenerative diseases. Lancet Neurol (2017) 16(4):323–32. 10.1016/S1474-4422(17)30037-6 28238712

[58] Del Tredici K Rüb U De Vos RA Bohl JR Braak H Where does Parkinson disease pathology begin in the brain? J Neuropathol Exp Neurol (2002) 61(5):413–26. 10.1093/jnen/61.5.413 12030260

[59] Luk KC Kehm V Carroll J Zhang B O’Brien P Trojanowski JQ Pathological α-synuclein transmission initiates Parkinson-like neurodegeneration in nontransgenic mice. Science (2012) 338(6109):949–53. 10.1126/science.1227157 23161999 PMC3552321

